# Rhythmic 24 h Variation of Core Body Temperature and Locomotor Activity in a Subterranean Rodent (*Ctenomys* aff. *knighti*), the Tuco-Tuco

**DOI:** 10.1371/journal.pone.0085674

**Published:** 2014-01-15

**Authors:** Patricia Tachinardi, José Eduardo Wilken Bicudo, Gisele Akemi Oda, Verónica Sandra Valentinuzzi

**Affiliations:** 1 Departamento de Fisiologia, Instituto de Biociências, Universidade de São Paulo, São Paulo, São Paulo, Brazil; 2 Centro Regional de Investigaciones Científicas y Transferencia Tecnológica de La Rioja (CRILAR-CONICET). Entre Ríos y Mendoza s/n, Anillaco, La Rioja, Argentina; Simon Fraser University, Canada

## Abstract

The tuco-tuco *Ctenomys* aff. *knighti* is a subterranean rodent which inhabits a semi-arid area in Northwestern Argentina. Although they live in underground burrows where environmental cycles are attenuated, they display robust, 24 h locomotor activity rhythms that are synchronized by light/dark cycles, both in laboratory and field conditions. The underground environment also poses energetic challenges (e.g. high-energy demands of digging, hypoxia, high humidity, low food availability) that have motivated thermoregulation studies in several subterranean rodent species. By using chronobiological protocols, the present work aims to contribute towards these studies by exploring day-night variations of thermoregulatory functions in tuco-tucos, starting with body temperature and its temporal relationship to locomotor activity. Animals showed daily, 24 h body temperature rhythms that persisted even in constant darkness and temperature, synchronizing to a daily light/dark cycle, with highest values occurring during darkness hours. The range of oscillation of body temperature was slightly lower than those reported for similar-sized and dark-active rodents. Most rhythmic parameters, such as period and phase, did not change upon removal of the running wheel. Body temperature and locomotor activity rhythms were robustly associated in time. The former persisted even after removal of the acute effects of intense activity on body temperature by a statistical method. Finally, regression gradients between body temperature and activity were higher in the beginning of the night, suggesting day-night variation in thermal conductance and heat production. Consideration of these day-night variations in thermoregulatory processes is beneficial for further studies on thermoregulation and energetics of subterranean rodents.

## Introduction

Daily, 24 h rhythmic variations in body temperature (Tb) are found in most mammals studied to date [1] and result from the association between daily rhythms of body heat “production” (endogenous increase of Tb) and heat “loss” (due to thermal conductance changes) [2], [3]. These might be adaptive for homoeothermic surface-dwellers, which face the daily challenge of maintaining their Tb within narrow limits, in an environment where ambient temperature varies on a day-night basis [4].

Subterranean rodents spend most of the time inside burrows in which the amplitude of the daily ambient temperature (Ta) and other environmental cycles are attenuated [5], [6]. The genus *Ctenomys* (Caviomorpha: Ctenomyidae), commonly known as tuco-tucos comprises more than 50 species occupying much of South America [7]. Besides one species that is known to be social [8], [9], most of them are solitary and emerge aboveground on a daily basis for foraging, therefore exposing themselves to surface Ta [10]–[12]. Several energetic studies have been carried out with *Ctenomys* species [13]–[17] in light of the thermoregulatory challenges of the underground environment, such as the high energy demands of digging, limited food quantity and quality and variable degrees of hypoxia/hypercapnia [18]–[20]. These studies are focused on daytime measurements and average values of thermoregulatory parameters. Our aim is to contribute towards exploring day-night variations of thermoregulatory functions, starting with the daily Tb rhythms, in these subterranean rodents.

Tuco-tucos of the species *Ctenomys* aff. *knighti* display robust circadian wheel running activity rhythms that are entrainable to light/dark (LD) cycles both in laboratory [21], [12] and field conditions [11]. In this sense, we hypothesized that they show robust and light-entrainable rhythmic patterns of Tb whose parameter values could contribute to a broader study of rhythmicity in subterranean rodents [22]. First, we verified the persistence of circadian Tb rhythms under constant conditions and tested for its photic entrainment. The timing association between Tb and locomotor activity rhythm was investigated along the entire protocol. Because vigorous activity elicited by the running wheel has already been shown to modulate rhythmicity [23], [24] we compared rhythmic parameters measured in the presence and in the absence of the wheel. Furthermore, because locomotor activity itself ensues acute Tb increases, a demasking method developed by Weinert and Waterhouse [25] was used to filter these increases. This method is based on the assumption that the acute effect of activity on Tb varies along the day, due to day-night variations in the thermal conductance of the body [3], [26], [27].

## Materials and Methods

### 1. Ethics statement

Trapping and experimental procedures were authorized by the Environmental Department of La Rioja (permits 028-10 and 062-08) and approved by the Ethics Committees of the Biosciences Institute of the University of São Paulo, Brazil (permit 153/2012) and of the Faculty of Veterinary Sciences of La Plata University, Argentina (permit 29-2-12). All the procedures followed the guidelines of the American Society of Mammalogists for the use of wild mammals in research [28].

### 2. Animals and housing conditions

Experiments were conducted in Anillaco (28° 48′ S; 66° 56′ W; 1350 m), located in the Argentinean province of La Rioja. Tuco-tucos used in this study were captured within a 3 km radius of Anillaco. Species identification of the animals found in this area is still undergoing (for details see [11]). In this paper they will be referred as *Ctenomys* aff. *knighti*.

Six adult females (#45, #46, #52, #100, #101 and #106; 128–176 g) and three adult males (#69, #97 and #98; 177–195 g) were housed individually in plastic cages equipped with running wheels (23 cm diameter, 10 cm wide, 1 cm between bars). Shredded paper was provided as nesting material and renewed weekly. Food (grass, carrot, sweet potato, rabbit pellets, oat, sunflower seeds) was provided *ad libitum* and replaced daily at random times. Water was not offered because subterranean rodents do not drink free water [29]. Each cage was placed inside a light-tight box, equipped with one incandescent red light bulb connected to a dimmer, which provided red dim light (1–5 lux), and one fluorescent bulb connected to a timer, which provided light intensity of 200–250 lux at cage lid level. To facilitate animal care, red dim light was kept “on” throughout all experiments, including the so-called “dark phase”. Animals were kept either under constant darkness (DD) or under a LD cycle with 12 hours of darkness followed by 12 hours of light (LD 12∶12). During the LD cycle the fluorescent light bulb was turned on at 07AM (local time, GMT -3). Relative humidity ranged from 30% to 60% and room temperature was maintained at 26±2°C, which is within the thermoneutral zone of other *Ctenomys* species [13]. Records of room temperature and relative humidity were taken every 15 minutes by HOBO U10/003 data loggers (Onset Computer Corporation, Bourne, MA).

### 3. Surgical procedures and data collection

To monitor core Tb and gross motor activity, telemetric transmitters (G2 E-Mitters, Mini-Mitter, Bend, OR) were implanted intraperitoneally. As surgical procedures were never performed in this species before, we developed a new protocol by adapting techniques and drug dosages used for other, similar-sized, rodent species. In summary, four animals (#45, #46, #52 and #69) were anesthetized with 100 mg/Kg of ketamine (Ketamina 50, Holliday-Scott S.A., Buenos Aires, Argentina) and 10 mg/Kg of xilazine hydrochloride (Kensol®, Avellaneda, Argentina). The remaining animals were anaesthetized with 200 mg/Kg of ketamine and 20 mg/Kg of acepromazine (Acedam, Holliday-Scott S.A., Buenos Aires) since this combination and dosage proved better efficiency and survival success. Tricotomy, local disinfection and carefully prepared surgery fields reduced infection risk. The frequent post-surgical removal of suture stitches by the animals was avoided using polyglicolic acid thread (the only material that did not generate allergic itching irritation), interrupted suture stitches (instead of continual), and home-made Elizabethan Collars during the hyperactive anesthesia recovery phase. The extremely thin abdominal muscular layer of this species required a small thread diameter (5-0 or 6-0). Hypothermia was avoided with thermal blankets (P010507, La-sure, São Paulo, Brazil). Immediately after surgery and in the following two days, tuco-tucos received a subcutaneous injection of antibiotic, enrofloxacin (Flotril® 2.5%, Schering-Plough, Rio de Janeiro, Brazil; 10 mg/Kg), and analgesic, flunixin meglumin (Banamine® Schering-Plough, Rio de Janeiro, Brazil; 2,5 mg/Kg). After surgery, animals were allowed three to five days of recovery before returning to the animal facility where the experiments took place.

Each cage was placed above receiver boards (ER 4000, Mini-Mitter, Bend, OR) connected to a computer where data was processed by the software VitalView (Mini-Mitter, Bend, OR). Running-wheel revolutions were recorded by the ArChron Data Acquisition System (Simonetta System, Universidad Nacional de Quilmes, Buenos Aires, Argentina). Recordings of all variables were made at 5-minute intervals.

### 4. Experiment 1

To verify endogenous, circadian rhythmicity and photic entrainment, six tuco-tucos (#54, #56, #52, #69, #97 and 98#) were kept under DD for 25–60 days. Next, animals were exposed to LD 12∶12 for 14–41 days followed by reestablishment of DD for 15–21 days. Different time intervals, in each condition for each animal, were due to individual differences in achievement of steady state entrainment and free-runs without aftereffects. (Table S1 shows the exact number of days to which each animal was exposed in each condition).

To verify the effect of running-wheel on rhythmic parameters, the running wheel was removed immediately after the previous protocol (while the animals were still in DD) and the tuco-tucos were exposed to the same series of conditions: 1) DD for 25–60 days; 2) LD for 14–41 days; 3) DD for 16–21 days (see Table S1). After wheel removal, we only relied on the intraperitoneal transmitter to monitor motor activity. Instead of measuring displacement, as was the case of the running wheels, this transmitter detects activity every time its angle relative to the receiver antenna changes.

### 5. Experiment 2

To investigate the acute effects of locomotor activity on Tb, we recorded the rhythms of three animals (#100, #101 and #106) kept under LD 12∶12. Initially these animals had access to the running wheel, for eight days. Then, the wheel was removed and the measurements continued for eight days.

### 6. Data analysis

#### Experiment 1

**Figure 1:**
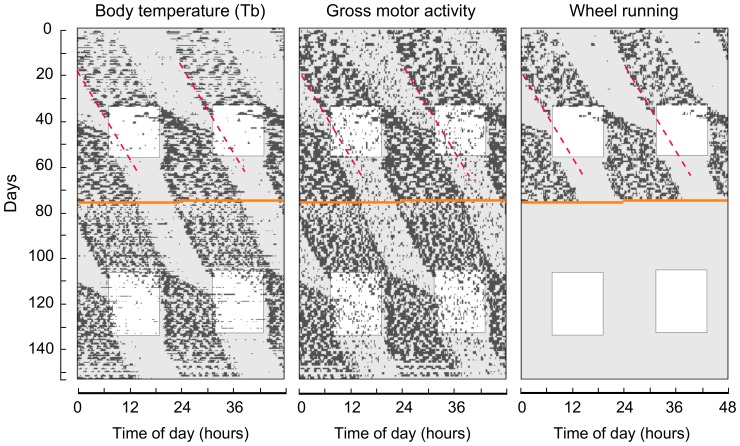
Free-running and synchronized rhythms, with and without running-wheels. Double-plotted actograms of Tb, gross motor activity and wheel running of a representative individual (#69). Black bars indicate the moments in which Tb rose above 36.2°C (left), gross motor activity rose above 50 counts (middle) and in which wheel running revolutions were detected (right). Gray and white backgrounds represent darkness and light hours, respectively. Orange line indicates the day in which the running wheel was removed. Red dashed lines indicate the onset of the free-running rhythms in the first DD exposure. Rhythms then synchronize to the LD cycle and upon reestablishment of DD, the phase of the onset is determined by the previously synchronized rhythm, not the one projected by the red line. This is indicative of entrainment of the circadian oscillator by the LD cycle, as opposed to masking of the output rhythms.

Data of all parameters were depicted in double-plotted actograms (see Fig. 1), using the software El Temps (Díez-Noguera, Universitat de Barcelona, Spain, 1999). To enhance the graphic output, the actograms of Tb are displayed only at values above a threshold determined for each animal. In this sense, a black bar was plotted across a 24-hours axis every time the temperature rose above the threshold. When temperature was lower than this value, a blank space was left. The gross motor activity actograms were constructed in a similar manner, with a black bar plotted every time the counts were higher than 50 per 5-min interval. Actograms were visually analyzed to estimate phase and rhythmic patterns. To determine periods in the different conditions, the chi-square periodogram analysis [30] was conducted, using 15-days data sets, with ClockLab software (Actimetrics, Evanston, IL).

To analyze range of oscillations and phase relationship between Tb and motor activity rhythms, waveforms were constructed, using the 10-day means for each time of the day. Phase relationship between temperature and activity rhythms was calculated using the onset of each waveform as the reference phase (calculated as phase relationship  =  onset Tb - onset wheel running). This onset was defined as the time when the waveform value exceeded the overall mean for at least one hour [31], [32]. The range of oscillation was calculated as the difference between the maximum and minimum mean values. Waveform depiction and analysis were performed with R version 2.11.1 [33].

#### Experiment 2

To identify the acute effects of locomotor activity on the Tb rhythm, linear regressions between gross E-mitter activity and Tb levels were made at different phases of the day, using data of LD synchronized rhythms, from animals with and without wheels. According to the employed demasking method [25], Tb at any time reflects the amount of activity that was integrated over a previous Integration Time (IT, in minutes) interval. IT was estimated based on the best correlation between activity and Tb levels. Tested IT values ranged from 10 to 60 minutes, in 5 min increments. Data series of Tb and activity integrated over previous IT intervals were constructed and each series was then divided into 2 h sections. Data obtained for each section, throughout all 16 days of measurements were grouped and IT chosen for the highest Pearson correlation, which corresponded to 20 minutes (Table S2 and Fig S1). Endogenous Tb values for null activity were extrapolated at each phase of the day through linear regressions of data corresponding to 3-hour intervals of the day. To investigate whether the effects of motor activity on Tb change during the day we calculated the average regression gradient (i.e. slope of the regression line) for 2-h sections. These gradients were calculated using activity integrated over 20 minutes. These calculations included data from animals in experiment 1 using, for each animal, 4 days with and 4 days without wheels, all under stable LD entrainment. To verify whether the differences in gradient along the day were significant, we used the non-parametric Friedman test to compare 4 sections of 6 hours each (22 h-04 h, 04 h-10 h, 10 h-16 h and 16 h-22 h). All analysis was performed with R version 2.11.1 [33].

## Results

### 1. Experiment 1

Body temperature, in all tuco-tucos, was rhythmic under DD, with periods slightly different from 24 hours (Fig. 1 and Table 1). Tb rhythm was strongly associated with motor activity rhythms, with identical period and stable phase relationship. When tuco-tucos were exposed to LD 12∶12, after a few transient days, all rhythms readily synchronized to the cycle. Highest temperatures and motor activity were concentrated in the dark phase.

**Table 1:** Periods of the Tb rhythm in DD and phase relationship between wheel running and Tb rhythms in LD.

	Period in DD		Phase relationship^1^ in LD
Animal	With wheel	Without wheel	Tb and wheel running
#45	24.2 h	24.2 h	32 min
#46	23.9 h	23.9 h	−35 min
#52	24.2 h	24.12 h	10 min
#69	24.2 h	24.2 h	−115 min
#97	24.3 h	24.2 h	30 min
#98	24.1 h	24.2 h	5 min

^1^ Phase relationship  =  onset Tb - onset wheel running

Upon reestablishment of DD (day 60), the phase of free-running rhythm was shown to be determined by the previous LD cycle (Fig. 1). This indicates that synchronization to the LD cycle occurred due to entrainment of the circadian oscillator. Under LD, phase relationship between wheel-running rhythm and Tb rhythm ranged between −115 and 32 minutes (Table 1).

Period of Tb and activity rhythms (Table 1) did not change significantly when the running-wheel was removed from the cage. Rhythms also synchronized to the LD cycle, with Tb remaining essentially high during the dark phase. Wheel removal resulted in a decrease of gross motor E-mitter activity in half of the individuals (Fig. S2).

**Figure 2:**
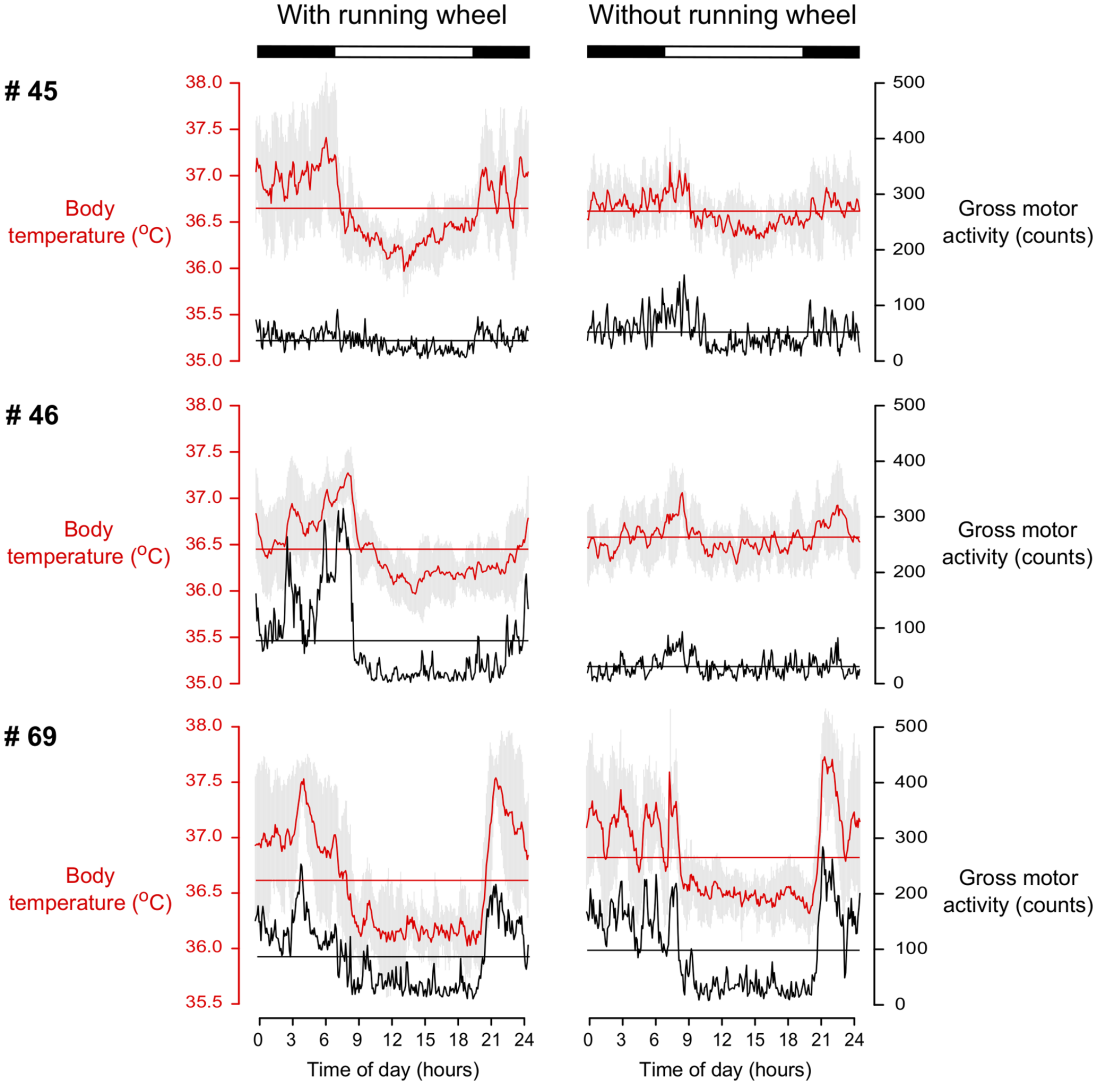
Temporal relationship between Tb and motor activity rhythms. Waveforms of Tb (red) and gross motor activity (black) rhythms of three individuals under LD 12∶12 (lights on at 07:00 am), with (left) and without (right) running-wheels. Each point represents the average of 10-day measures for the corresponding time of the day. Horizontal lines indicate the mean of the total values obtained for each variable and vertical gray line represent the standard deviation for Tb for each time over the 10 days.

In four animals, the mean value of Tb was higher (0.13–0.2°C) after wheel removal and in two it decreased slightly (0.03–0.1°C) (Fig. 2 and Table S3). The range of oscillation values of Tb rhythms reported when individuals had running wheels were closer to the predicted values for similar sized rodents [2] (Table 2) and consistently larger than those reported when the wheel was removed, and this trend was observed in five out of six of the experimental individuals used. Only individual #97 showed an inverse situation.

**Table 2:** Range of oscillation of Tb rhythms synchronized to the LD cycle.

Animal	Mass	With wheel	Without wheel	Predicted^1^
#45	152 g	1.44°C	0.82°C	1.770°C
#46	176 g	1.31°C	0.77°C	1.720°C
#52	146 g	1.03°C	0.95°C	1.784°C
#69	177 g	1.52°C	1.40°C	1.718°C
#97	195 g	0.80°C	0.96°C	1.685°C
#98	190 g	1.37°C	1.07°C	1.694°C

^1^ Based on the equation ***log RT  =  log 4.762***– ***0.197 log M_b_*** (Aschoff, 1982), where ***RT*** is range of oscillation and ***M_b_*** is body mass.

### 2. Experiment 2

**Figure 3:**
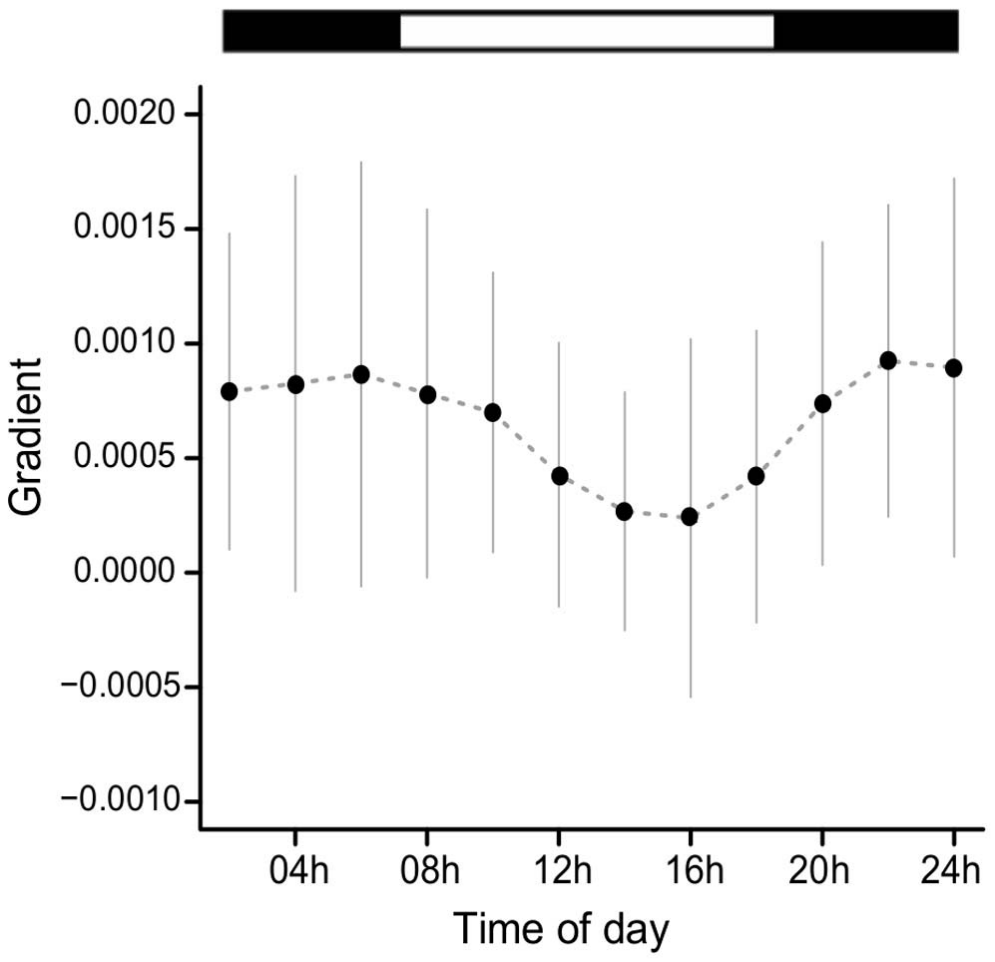
Gradients of linear regression of Tb and motor activity in different times of the day. Average gradients of the linear regression model of Tb and motor activity (integrated over 20 min) of nine animals under an LD cycle. Regressions were performed, for each animal, in 2h-windows, using data from 4 days when they had access to a running wheel and from 4 days when the wheel was absent. Gray lines indicate the standard deviation. The bar above the graph represents the light (white)/dark (black) cycle.

**Figure 4:**
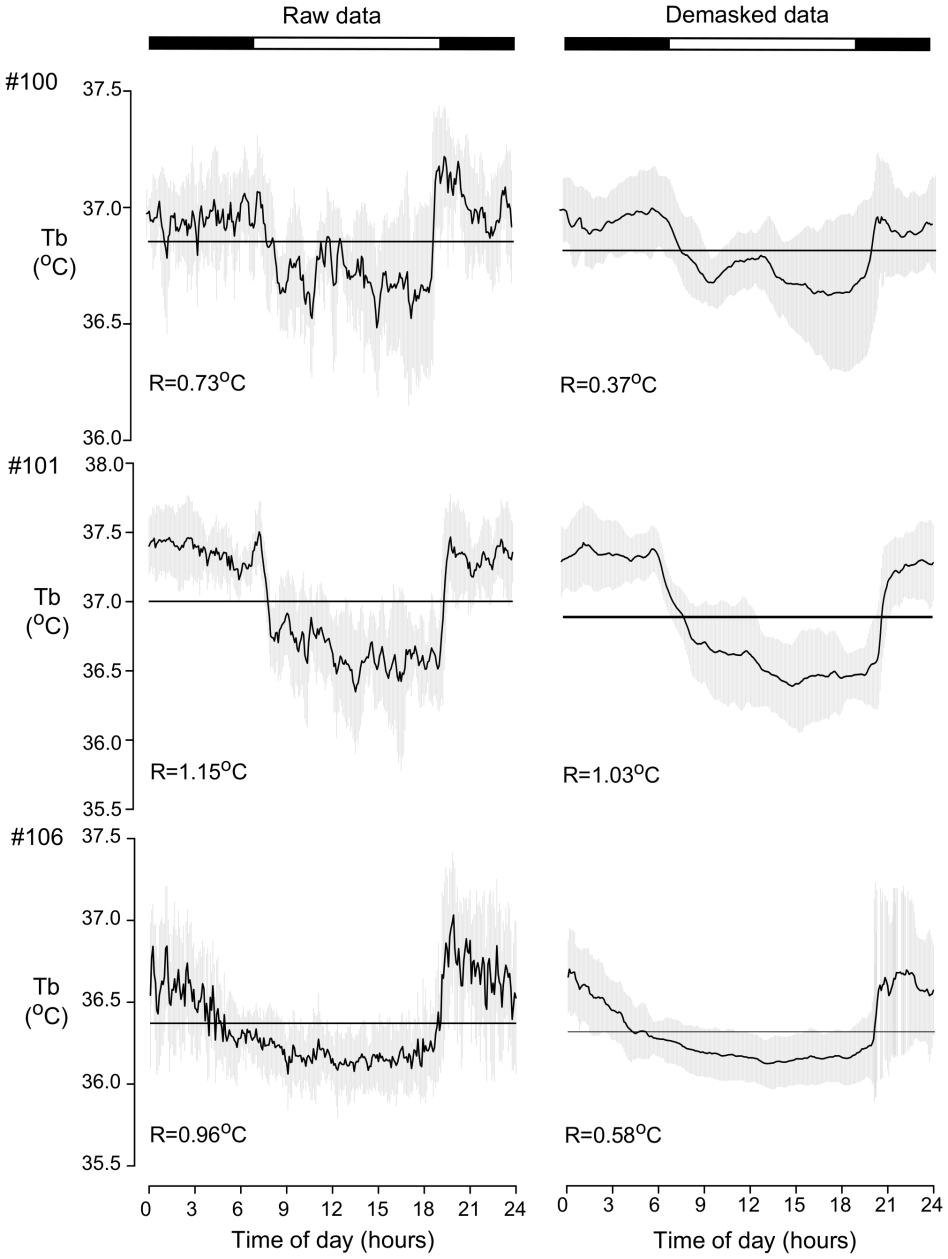
Comparison of the daily Tb rhythm before and after the demasking treatment. Waveforms of raw (left) and demasked (right) data from three animals with access to a running wheel under LD 12∶12 cycle. Each point represents the average of 8-day measures for the corresponding time of the day. Horizontal lines indicate the mean of the total values obtained for raw and demasked Tb and vertical gray line represent the standard deviation for Tb for each time over the 8 days. Above each waveform are the range of oscillation values. The numbers on the left are the codes for each animal.

The regression gradients between activity and Tb were higher during darkness hours than light hours (Fig. 3). Friedman test indicated that the differences in gradient during the day were significant (p<0.05). Demasking of Tb rhythm consisted of subtracting the amount of Tb due to the acute effects of locomotor activity, at each hour of the day. A comparison between raw Tb data and demasked data is shown in Fig. 4. The demasked curve is clearly smoother than the raw data, indicating that several acute effects of activity bouts were filtered. Periods and peak phases did not change after demasking and range of oscillation changes are shown in Fig. 4.

## Discussion

All tuco-tucos exhibited circadian rhythms of Tb. These rhythms were closely associated in time with the motor activity rhythms and showed a nocturnal pattern under the 12∶12 LD cycle. Synchronization to LD resulted from the entrainment of the oscillator rather than by a direct reaction to light (masking), as evidenced by transient cycles upon LD establishment and the maintenance of the phase determined by LD upon reestablishment of DD (Fig.1) [34].

The circadian range of oscillation of Tb under a LD cycle (from 0.8 to 1.4°C) was slightly lower than that predicted for non-primate mammals according to the equation given by Aschoff [2], which relates the range of oscillation in Tb and body mass (Table 2). This range of oscillation was also lower compared with that of other similar-sized rodents that are dark-active under 24 h LD cycles (e.g., 2°C and 2.9°C in Long-Evans rat and Syrian hamster, respectively) [35]. On the other hand, the mean Tb values (from 36.35 to 36.81°C) were similar to the ones reported for other *Ctenomys* species (36.1°C and 37.3°C, *C. talarum* and *C. australis,* respectively) [13], but higher compared to other subterranean rodents (e.g., 35.1°C and 35.0°C for *Heliophobius argenteocinereus* and *Fukomys damarensis, respectively*) [20], [36] at thermoneutrality. It is similar to the mean interspecific Tb of 36.9°C, reported by Arends and McNab [37] calculated for 30 caviomorph rodent species from several families and habitats. Interestingly, Caviomorph rodents have a higher basal metabolic rate than other mammals of the same body mass [37].

Several studies have indicated that rhythmic parameters may be modulated by the running-wheel, a device that has traditionally been employed for chronobiological measurements. Rodents are prone to run vigorously on running-wheels [38], [39] and the ensuing high activity levels might feedback on the circadian oscillator [40]. In contrast to the findings in hamsters, rats and mice [41], [42], [23], vigorous running on the wheel did not change rhythmic parameters such as the circadian period in the tuco-tuco (Fig.1). Furthermore, changes in the phase of LD synchronized rhythms, such as the dramatic switch from a nocturnal to diurnal pattern reported for Nile grass rats (*Arvicanthis niloticus*) [24] and the degu (*Octodon degus*) [43] was not observed in tuco-tucos upon wheel removal (Fig.1). This switch is not uncommon and has already been reported in *Ctenomys* aff. *knighti* under a different context [11]. All tuco-tucos ran vigorously on the wheels, but data from the implanted E-Mitters showed that removal of the wheel did not decrease activity levels in half of the animals (Figure S2). This discrepancy may be related to inter-individual differences in the expression of new behaviors upon wheel removal, such as climbing [44] and digging-like behavior. Notwithstanding, a trend for range of oscillation decrease was observed upon wheel removal (Table 2). Reduced range of oscillation of the Tb rhythm after running wheel removal was reported for hamsters [45], [46] and the subterranean rodent *Heterocephalus glaber* [47]. When animals have access to running wheels, higher Tb during the active phase possibly occur in part due to the direct contribution of heat generated by muscular activity. This hypothesis is supported by the fact that shortening of the range of oscillation occurred mainly due to a decrease in maximal values, rather than to an increase in minimum values [48].

The best correlation between Tb and activity level increases were found for IT = 20 min. This means that it takes an average 20 min for an amount of muscular activity to increase Tb, due to the time it takes for muscle heat to be distributed through the body and to its thermal capacity [25]. The regression gradients between Tb and activity levels were on average higher during the dark phase of the light/dark cycle (Fig.3), indicating that the same amount of activity ensues higher Tb increase during the night than during the day [26]. This may be due to a daily variation in body conductance, with less exercise heat dissipated during the dark phase and due to the daily variation in heat production [25]. This is similar to the findings in rats [49] and opposite to what was found in mice [25], indicating interspecies differences among nocturnal species. Higher body conductance that facilitates heat dissipation during the day might explain the tiny Tb increment elicited by simulated, vigorous digging activity in tuco-tucos, performed during day-time hours [15]. Defense of low metabolic rates constitutes a common strategy for energy savings in subterranean rodents [13] and these have been reported for other *Ctenomys* species [16]. Lowered conductance due to higher peripheral vasoconstriction during the night might contribute to energy saving and higher Tb during the night in these nocturnal species. Finally, Gordon and Yang [27] have revealed the further complexity of the temporal correlations between Tb and locomotor activity by showing their dependence also on sex, reproductive cycle stage and average ambient temperatures.

To which extent precise Tb rhythms improve the animal's fitness in their natural habitat is an issue still to be investigated, but some speculations can be made. Tb rhythms may be important for thermoregulation in an environment with daily Ta cycles, considering they are a result of the association of the rhythms of heat loss and production [2]. One would think this is not the case for subterranean rodents, which live in tunnels where the daily temperature changes are rather small. Body temperature studies in different species of strictly subterranean rodents from Asia and Africa reported great inter-individual variability of Tb rhythmicity, with the occurrence of nocturnal, diurnal, crepuscular and even arrhythmic individuals within the same species [50], [22], [51]. This is in sharp contrast to what is exhibited by the tuco-tucos, in which Tb is always rhythmic and nocturnal under laboratory conditions. These results reinforce the statement that *C.* aff *knighti* displays much less polymorphism in rhythmic patterns than other subterranean rodents [21]. Some morphological features, such as the size of the eye and structure of the retina, also distinguish ctenomyids from other subterranean rodents [52]. Remarkably, tuco-tucos forage aboveground [10], [11] more frequently than other strictly subterranean rodents [53], [47] and it has been shown that these surface excursions ensure photic entrainment of their circadian oscillators [12]. Thus, they are more exposed to external conditions, which could have been a selective pressure for the maintenance of rhythmicity. Another adaptive value of the Tb rhythm may be that it possibly plays a major role as an internal synchronizing cue for peripheral oscillators [54]. This is of great importance in order to maintain internal temporal order, which is vital even for organisms living mostly in underground environments, such as the tuco-tucos. Further studies on whether tuco-tucos are indeed rhythmic in the field will certainly provide insights on the meaning of biological rhythms for these animals, which live in such a peculiar environment.

## Supporting Information

Figure S1 **Average correlation coefficients for several integration times (IT**)**.** Averages were calculated by Pearson method, from data of seven individuals maintained under an LD 12∶12 cycle. Three individuals were used in Experiment 2 and four others were maintained in the same conditions but were not used in further experiments.
(TIFF)

Figure S2 **Means of the daily total gross motor activity over 10 days (in LD) in the presence (gray) and absence (white) of running wheels.** Black vertical lines show the standard deviation. Asterisks indicate significant difference between the two conditions (T-test p<0.05).
(TIFF)

Table S1 **Information about the individuals used in this study and number of days each animal spent under each condition.**
(DOCX)

Table S2 **Average Pearson correlation coefficients for several integration times (IT) in each 2 h window.** Averages were calculated from data of seven individuals maintained under an LD 12∶12 cycle. The highest coefficient for each 2h-window are highlighted in red. Except for window 8–10 h and 16–18 h, higher correlations were found for IT = 20 min.
(DOCX)

Table S3 Parameters of the Tb rhythm with and without a running wheel.
(DOCX)
