# Supplementary figures and images for: Rhythmic 24 h Variation of Core Body Temperature and Locomotor Activity in a Subterranean Rodent (Ctenomys aff. knighti), the Tuco-Tuco
Source: PLoS One. 2014 Jan 15;9(1):e85674. doi: 10.1371/journal.pone.0085674 (PMC3893220; doi:10.1371/journal.pone.0085674)

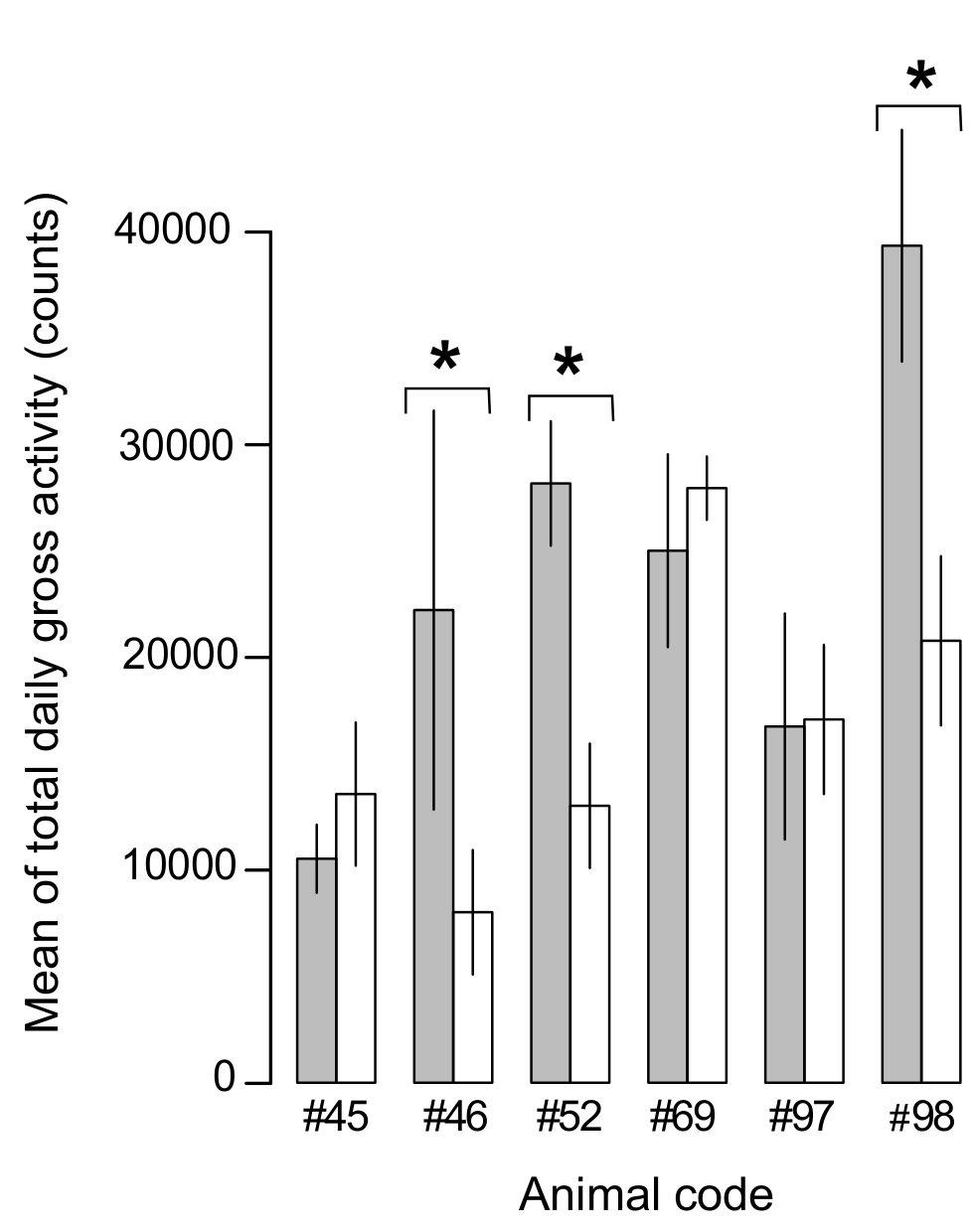

Supplement: Figure S2 — Means of the daily total gross motor activity over 10 days (in LD) in the presence (gray) and absence (white) of running wheels. Black vertical lines show the standard deviation. Asterisks indicate significant difference between the two conditions (T-test p<0.05). (TIFF) [file pone.0085674.s002.tiff]
