# Supplementary material for: Rhythmic 24 h Variation of Core Body Temperature and Locomotor Activity in a Subterranean Rodent (Ctenomys aff. knighti), the Tuco-Tuco
Source: PLoS One. 2014 Jan 15;9(1):e85674. doi: 10.1371/journal.pone.0085674 (PMC3893220; doi:10.1371/journal.pone.0085674)
Supplement: Table S1 — Information about the individuals used in this study and number of days each animal spent under each condition. (DOCX) [file pone.0085674.s003.docx]

**Table S1.** Information about the individuals used in this study and number of days each animal spent under each condition.

|  |  |  | **Experiment 1** | | | | | | **Experiment 2** | |
| --- | --- | --- | --- | --- | --- | --- | --- | --- | --- | --- |
|  |  |  | **Wheel** | | | **No Wheel** | | |  | |
| **Animal** | **Sex** | **Mass (g)** | **1^st^ DD** | **LD** | **2^nd^ DD** | **1^st^ DD** | **LD** | **2^nd^ DD** | **Wheel** | **No wheel** |
| #45 | ♀ | 152 | 44 days | 14 days | 16 days | 28 days | 30 days | 15 days | - | - |
| #46 | ♀ | 176 | 44 days | 14 days | 16 days | 28 days | 41 days | 15 days | - | - |
| #52 | ♀ | 146 | 44 days | 14 days | 16 days | 28 days | 41 days | 15 days | - | - |
| #69 | ♂ | 177 | 25 days | 23 days | 15 days | 31 days | 28 days | 15 days | - | - |
| #97 | ♂ | 195 | 39 days | 41 days | 21 days | 31 days | 21 days | 15 days | - | - |
| #98 | ♂ | 190 | 60 days | 30 days | 21 days | 31 days | 21 days | 15 days | - | - |
| #100 | ♀ | 128 | - | - | - | - | - | - | 8 days | 8 days |
| #101 | ♀ | 153 | - | - | - | - | - | - | 8 days | 8 days |
| #106 | ♀ | 135 | - | - | - | - | - | - | 8 days | 8 days |
