# Supplementary material for: Rhythmic 24 h Variation of Core Body Temperature and Locomotor Activity in a Subterranean Rodent (Ctenomys aff. knighti), the Tuco-Tuco
Source: PLoS One. 2014 Jan 15;9(1):e85674. doi: 10.1371/journal.pone.0085674 (PMC3893220; doi:10.1371/journal.pone.0085674)
Supplement: Table S2 — Average Pearson correlation coefficients for several integration times (IT) in each 2 h window. Averages were calculated from data of seven individuals maintained under an LD 12∶12 cycle. The highest coefficient for each 2h-window are highlighted in red. Except for window 8–10 h and 16–18 h, higher correlations were found for IT = 20 min. (DOCX) [file pone.0085674.s004.docx]

**Table S2.** Average Pearson correlation coefficients for several integration times (IT) in each 2h window. Averages were calculated from data of seven individuals maintained under an LD 12:12 cycle. The highest coefficient for each 2h-window are highlighted in red. Except for window 8-10h and 16-18h, higher correlations were found for IT=20min.

| **IT** | **0-2h** | **2-4h** | **4-6h** | **6-8h** | **8-10h** | **10-12h** | **12-14h** | **14-16h** | **16-18h** | **18-20h** | **20-22h** | **22-24h** |
| --- | --- | --- | --- | --- | --- | --- | --- | --- | --- | --- | --- | --- |
| 10 | 0.40 | 0.37 | 0.28 | 0.17 | 0.14 | 0.08 | -0.02 | 0.08 | 0.29 | 0.56 | 0.48 | 0.42 |
| 15 | 0.44 | 0.39 | 0.31 | 0.18 | 0.16 | 0.10 | -0.01 | 0.09 | 0.32 | 0.58 | 0.51 | 0.45 |
| 20 | **0.51** | **0.48** | **0.39** | **0.23** | 0.23 | **0.17** | **0.53** | **0.14** | 0.34 | **0.63** | **0.57** | **0.53** |
| 25 | 0.46 | 0.39 | 0.34 | 0.18 | 0.21 | 0.13 | 0.01 | 0.10 | 0.35 | 0.61 | 0.53 | 0.47 |
| 30 | 0.46 | 0.38 | 0.36 | 0.19 | 0.23 | 0.13 | 0.01 | 0.01 | **0.35** | 0.61 | 0.52 | 0.47 |
| 35 | 0.46 | 0.37 | 0.36 | 0.19 | 0.24 | 0.13 | 0.02 | 0.09 | 0.35 | 0.60 | 0.52 | 0.47 |
| 40 | 0.45 | 0.36 | 0.37 | 0.19 | 0.24 | 0.13 | 0.02 | 0.08 | 0.34 | 0.59 | 0.51 | 0.46 |
| 45 | 0.44 | 0.35 | 0.37 | 0.19 | 0.25 | 0.12 | 0.01 | 0.07 | 0.34 | 0.58 | 0.50 | 0.45 |
| 50 | 0.42 | 0.35 | 0.36 | 0.18 | **0.25** | 0.10 | 0.00 | 0.06 | 0.33 | 0.57 | 0.49 | 0.43 |
| 55 | 0.41 | 0.34 | 0.35 | 0.18 | 0.25 | 0.09 | -0.01 | 0.05 | 0.32 | 0.56 | 0.48 | 0.42 |
| 60 | 0.39 | 0.33 | 0.34 | 0.18 | 0.24 | 0.08 | -0.02 | 0.03 | 0.32 | 0.55 | 0.47 | 0.40 |
