# Supplementary material for: Rhythmic 24 h Variation of Core Body Temperature and Locomotor Activity in a Subterranean Rodent (Ctenomys aff. knighti), the Tuco-Tuco
Source: PLoS One. 2014 Jan 15;9(1):e85674. doi: 10.1371/journal.pone.0085674 (PMC3893220; doi:10.1371/journal.pone.0085674)
Supplement: Table S3 — Parameters of the Tb rhythm with and without a running wheel. (DOCX) [file pone.0085674.s005.docx]

**Table S3.** Parameters of the Tb rhythm with and without a running wheel.

|  |  | **Mean (^o^C)** | | |  | **Maximum (^o^C)** | | |  | **Minimum (^o^C)** | | |  | **Range of oscillation (^o^C)** | | |
| --- | --- | --- | --- | --- | --- | --- | --- | --- | --- | --- | --- | --- | --- | --- | --- | --- |
| **Animal** |  | **With wheel** | **Without wheel** | **Difference** |  | **With wheel** | **Without wheel** | **Difference** |  | **With wheel** | **Without wheel** | **Difference** |  | **With wheel** | **Without wheel** | **Difference** |
| **#45** |  | 36.65 | 36.62 | -0.03 |  | 37.41 | 37.14 | 0.48 |  | 35.97 | 36.32 | 0.35 |  | 1.44 | 0.82 | -0.62 |
| **#46** |  | 36.45 | 36.58 | 0.13 |  | 37.27 | 37.06 | -0.21 |  | 35.97 | 36.29 | 0.32 |  | 1.31 | 0.77 | -0.54 |
| **#52** |  | 36.35 | 36.25 | -0.1 |  | 37.96 | 37.89 | -0.07 |  | 36.93 | 36.94 | 0.01 |  | 1.03 | 0.95 | -0.08 |
| **#69** |  | 36.61 | 36.81 | 0.20 |  | 37.54 | 37.71 | 0.17 |  | 36.02 | 36.31 | 0.29 |  | 1.52 | 1.4 | -0.12 |
| **#97** |  | 36.54 | 36.73 | 0.19 |  | 36.94 | 37.29 | 0.35 |  | 36.13 | 36.32 | 0.19 |  | 0.8 | 0.96 | 0.16 |
| **#98** |  | 36.59 | 36.75 | 0.16 |  | 37.34 | 37.37 | 0.03 |  | 35.97 | 36.3 | 0.33 |  | 1.37 | 1.07 | -0.29 |
